# Neuroprotective Effects of Low-Dose Graphenic Materials on SN4741 Embryonic Stem Cells Against ER Stress and MPTP-Induced Oxidative Stress

**DOI:** 10.3390/ijms26188821

**Published:** 2025-09-10

**Authors:** David Vallejo Perez, Monica Navarro, Beatriz Segura-Segura, Rune Wendelbo, Sara Bandrés-Ciga, Miguel A. Arraez, Cinta Arraez, Noela Rodriguez-Losada

**Affiliations:** 1 Neurosurgery Unit, Biomedicine Research Institute of Malaga (IBIMA), Department of Neurosurgery, Málaga Regional University Hospital, 29010 Malaga, Spain; david.vallejo.sspa@juntadeandalucia.es (D.V.P.); marraezs@uma.es (M.A.A.); cinta.arraez@gmail.com (C.A.); 2 Catedra de Fisiologia, Facultad de Farmacia y Bioquimica, Universidad de Buenos Aires, Buenos Aires 1113, Argentina; mnavarro@ffyb.uba.ar; 3 Instituto de Química y Metabolismo del Farmaco (IQUIMEFA), CONICET-Universidad de Buenos Aires, Buenos Aires 1113, Argentina; 4 Vaccine Research Department, Fundación para el Fomento de la Investigación Sanitaria y Biomédica de la Comunidad Valenciana (FISABIO), 46024 Valencia, Spain; beatriz.segura@fisabio.es; 5 CIBER de Epidemiología y Salud Publica, Instituto de Salud Pública de Valencia, 46024 Valencia, Spain; 6 Shift Materials AS, 0663 Oslo, Norway; rw@graphenebatteries.no; 7 Center for Alzheimer’s and Related Dementias (CARD), National Institute on Aging and National Institute of Neurological Disorders and Stroke, National Institutes of Health, Bethesda, MD 20892, USA; sara.bandresciga@nih.gov; 8 Department of Surgical Specialties, Biochemistry and Immunology, School of Medicine, University of Málaga, 29071 Malaga, Spain; 9 Department of Neurosurgery, Vithas Hospital, 29016 Malaga, Spain; 10 Faculty of Science Education, Department of Didactic Science Education, University of Malaga, 29010 Malaga, Spain

**Keywords:** parkinson’s disease, neuroprotection, graphene oxide, graphene reduced, er-stress, neurodegenerative diseases

## Abstract

In this study, we explore the neuroprotective and modulatory potential of graphenic materials (GMs) in terms of the maturation of dopaminergic neurons and their capacity to counteract the cellular stress induced by toxins such as MPP^+^ (1-methyl-4-phenylpyridinium) and Tunicamycin. We found that GMs promote significant morphological changes in neuronal cells after prolonged exposure, enhancing both differentiation and cellular adhesion. Through structural analysis, we unveiled a complex organization of GMs and a marked upregulation of tyrosine hydroxylase (TH), a key marker of mature dopaminergic neurons. Under oxidative stress induced by MPP^+^, GMs significantly reduced the release of lactate dehydrogenase (LDH), indicating protection against mitochondrial damage. Moreover, GMs substantially decreased the levels of α-synuclein (α-Syn), a protein closely associated with neurodegenerative disorders such as Parkinson’s disease. Notably, partially reduced graphene oxide (PRGO) and fully reduced graphene oxide (FRGO) films were particularly effective at reducing α-Syn-associated toxicity compared to positive controls. Under conditions of endoplasmic reticulum (ER) stress triggered by Tunicamycin, GMs—especially PRGO microflakes—modulated the unfolded protein response (UPR) pathway. This effect was evidenced by the increased expression of BIP/GRP78 and the decreased phosphorylation of stress sensors such as PERK and eIF2α; this suggests that a protective role is played against ER stress. Additionally, GMs enhanced the synthesis of Torsin 1A, a chaperone protein involved in correcting protein folding defects, with PRGO microflakes showing up to a fivefold increase relative to the controls. Through the cFos analysis, we further revealed a pre-adaptive cellular response in GM-treated cells exposed to MPP^+^, with PRGO microflakes inducing a significant twofold increase in cFos expression compared to the positive control, indicating partial protection against oxidative stress. In conclusion, these results underscore GMs’ capacity to modulate the critical cellular pathways involved in oxidative, mitochondrial, and ER stress responses, positioning them as promising candidates for future neuroprotective and therapeutic strategies.

## 1. Introduction

Parkinson’s disease (PD) is a complex neurodegenerative disorder characterized by the progressive loss of dopaminergic neurons in the substantia nigra pars compacta (SNpc). The causes of PD are multifactorial, involving a combination of genetic, environmental, and pathophysiological factors [1]. PD is characterized by the selective degeneration of dopaminergic neurons in the substantia nigra pars compacta, leading to motor dysfunction, cognitive decline, and other debilitating symptoms [2]. A distinguishing feature of PD is the presence of intracellular inclusions known as Lewy bodies [3]. These consist of insoluble aggregates of misfolded alpha-synuclein protein (α-Syn). Recent studies have linked α-Syn pathology to dysfunction in the endoplasmic reticulum (ER) [4]. The aggregation of α-Syn leads to the fragmentation of the ER, impairing its ability to properly fold proteins and clear misfolded proteins, which triggers intracellular stress [5].

In recent studies, elevated levels of α-Syn have been associated with endoplasmic reticulum (ER) dysfunction. These aggregates induce ER fragmentation, reducing the ER’s capacity for protein folding and processing misfolded proteins, thereby generating intracellular stress. Elevated levels of α-Syn impair the unfolded protein response (UPR), leading to chronic ER stress that ultimately results in cellular death [6,7]. The unfolded protein response and the modulation of stress-responsive proteins, such as BIP/GRP78 and Torsin 1A, represent critical mechanisms in the cellular adaptation to ER stress [8,9]. ER stress, triggered by agents such as Tunicamycin, leads to the accumulation of misfolded proteins and the activation of the UPR, a signaling cascade used to restore proteostasis.

At the cellular level, PD is associated with increased production of reactive oxygen species (ROS) [10]. Deficiencies in mitochondrial complex I of the electron transport chain are major contributors to neuronal apoptosis and are considered one of the primary sources of ROS in PD [11]. Reduced complex I activity is well documented in PD patients [12]. Furthermore, complex I inhibitors, such as 1-methyl-4-phenyl-1,2,3,6-tetrahydropyridine (MPTP) and rotenone, exhibit selective toxicity towards dopaminergic neurons, reinforcing the role of mitochondrial dysfunction in the pathogenesis of PD [13].

Another key indicator in the study of PD pathophysiology is lactic acid, which is produced by the activity of the lactate dehydrogenase (LDH) enzyme [14]. Elevated LDH levels in PD are not simply markers of cellular damage, but reflect significant disturbances in cerebral bioenergetic metabolism driven by mitochondrial dysfunction, oxidative stress, and impaired redox homeostasis. In this context, serum LDH emerges as an accessible biomarker of systemic cellular stress, with potential utility in monitoring disease progression [15]. The dysregulated expression of LDH isoforms (LDHA/LDHB) and the disruption of the astrocyte–neuron lactate shuttle highlight the central role of metabolic dysregulation in Parkinson’s disease. Additionally, mutations in the PARKIN gene (PARK2), associated with familial forms of PD, have been linked to mitochondrial instability and increased glycolytic flux, indirectly promoting the overexpression of LDHA [16]. Similarly, alpha-synuclein protein (encoded by the SNCA gene), which aggregates in Lewy bodies, disrupts mitochondrial function and may induce energetic stress, favoring a shift towards glycolytic metabolism and increasing reliance on LDH. LDH-A drives lactate accumulation, which promotes the apoptosis of dopaminergic neurons. Notably, abnormally elevated lactate levels have been reported in the cerebrospinal fluid (CSF) of patients with advanced Parkinson’s disease [17]. Together, these findings establish LDH activity and extracellular lactate levels as key markers of dopaminergic damage and critical factors in regulating the progression of PD. Another critical aspect regarding neuroprotection is related to the modulation of immediate early gene (IEG) responses such as cFos expression, which is pivotal in regulating cellular adaptation to external stimuli like cellular stress [18,19,20,21].

Over the past decade, graphene (G) and graphene oxide (GO), discovered by Novoselov et al. (2004) [22], have emerged as promising nanomaterials to be tested as scaffolds for electroconductive tissues, such as neurons, due to their biocompatible physical and chemical properties. Graphene is a planar nanocomposite consisting of hexagonal lattice-structured carbon rings with sp^2^-hybridized carbons that confer excellent electrical conductivity [22]. Recent studies have demonstrated that GMs can stimulate neuronal maturation and functional recovery, underscoring their potential in regenerative medicine [23,24]. Graphenic materials (GMs), including graphene oxide (GO), partially reduced graphene oxide (PRGO), and fully reduced graphene oxide (FRGO), exhibit excellent biocompatibility, tunable surface chemistry, and the ability to modulate cellular behavior [25,26]. These properties mean that GMs are promising candidates for promoting neuronal maturation, protecting against neurotoxic insults, and supporting functional recovery in neurodegenerative conditions [27]. Our previous studies demonstrated that GMs can enhance the structural and functional maturation of dopaminergic neurons in vitro. Specifically, PRGO and FRGO films have been shown to support the development of elongated, axon-like processes and promote the expression of key markers associated with dopaminergic lineage commitment, such as tyrosine hydroxylase (TH) and GIRK-2 [25]. We hypothesize that GMs may have neuroprotective effects on neurons that are damaged by oxidative stress caused by MPTP. Furthermore, we questioned whether this protective effect could be mediated by reducing endoplasmic reticulum (ER) stress [28] by decreasing the levels of α-synuclein (α-Syn) and lactate dehydrogenase (LDH), which are key biomarkers associated with the progression and severity of Parkinson’s disease.

## 2. Results

### 2.1. Maturation and Integrity of DA Phenotype in Culture with GMs

**Figure 1:**
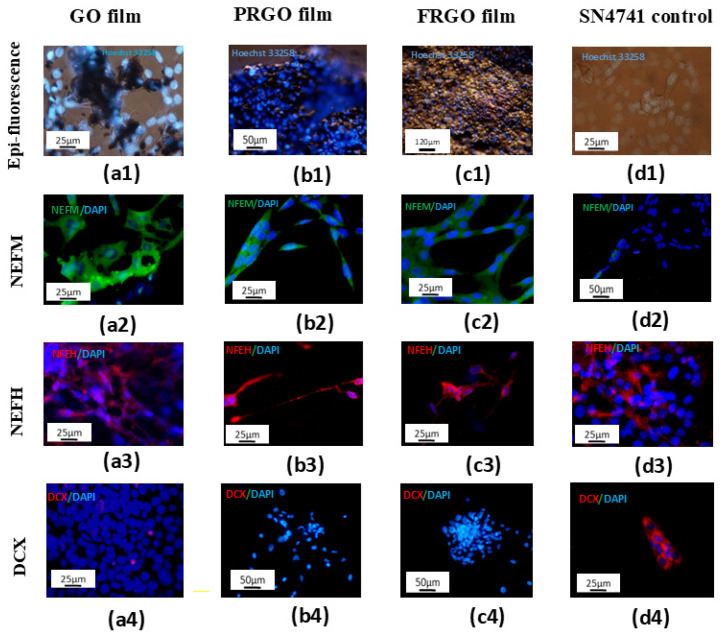
GMs promote maturation and biocompatibility in SN4741 cell cultures (7 days in culture). Nuclear staining with Hoechst 3258 (in vivo) shows no apoptotic bodies in adherent cells cultured on GM substrates. (**a1**) GO film; (**b1**) PRGO film; (**c1**) FRGO film; (**d1**) control. Merged images display neurofilament-M (NEFM) (green)/DAPI (blue) in (**a2**–**d2**) and neurofilament-H (NEFH) (red)/DAPI (blue) in (**a3**–**d3**) Immature cell marker DCX (red)/DAPI (blue) is shown in (**a4**–**d4**). Scale bars: 25 µm (**a1**,**d1**,**a2**–**c2**,**a3**,**d3**,**a4**,**d4**); 50 µm (**b1**,**d2**,**b4**,**c4**); 120 µm (**c1**).

The differentiating capacity of GMs was further evidenced when we carried out immunostaining for a neuron-specific structural protein and neurofilament-H (Figure 1(a2–d2)). GM-treated cultures, particularly those with FRGO film and PRGO film as bioscaffolds, displayed organized neurofilament distribution indicative of structural integrity. The PRGO film notably supported SN4741 cells with elongated, axon-like processes. In contrast, the GO film and control groups showed weak or irregular cytoplasmic staining. The absence of Doublecortin confirmed the maturation potential of GMs used as an immature neuron marker (DCX, Figure 1(a3–c3)) in the PRGO film and FRGO film cultures. Conversely, the GO film exhibited low DCX expression, while the control SN4741 cells (Figure 1(d3)) displayed strong DCX positivity, underscoring GO film’s limited capacity to promote neuronal maturation.

**Figure 2:**
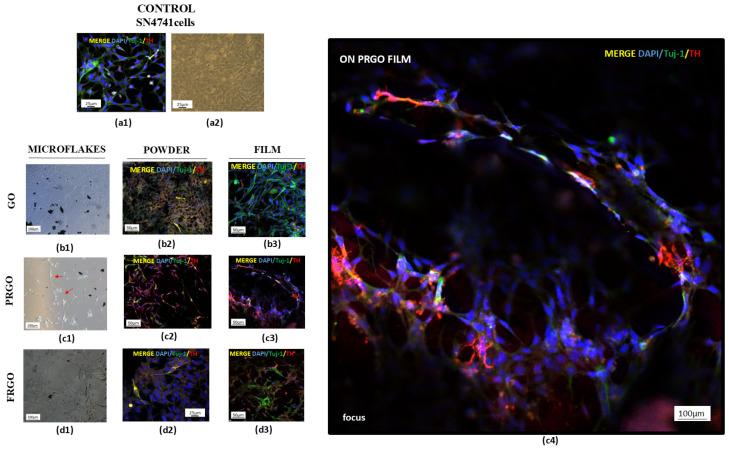
Maturation of SN4741 cells on GM microflakes. Untreated control cells showing Tuj-1 (green)/TH (red)/DAPI (blue) and brightfield morphology. (**a1**,**a2**). Brightfield images of cells on GO (**b1**), PRGO (**c1**), and FRGO (**d1**) microflakes. Merged Tuj-1/TH/DAPI on (**b2**–**d2**) powder substrates and (**b3**–**d3**) films ((**c4**) focus, cells on PRGO film). Representative images are presented with magnification: 4× (**c1**,**d1**); 20× (**a1**,**a2**,**b1**–**b3**,**c2**,**c3**,**d2**,**d3**); 100× (**c4**).

The results of the maturation of SN4741 cells are displayed in Figure 2. In long-term cultures exposed to GMs, SN4741 cells displayed neuronal-like morphological alterations, evidenced by the coexpression of Tuj-1 (a pan-neuronal marker) and TH as dopaminergic mature markers, consistent with previous findings [26].

**Figure 3:**
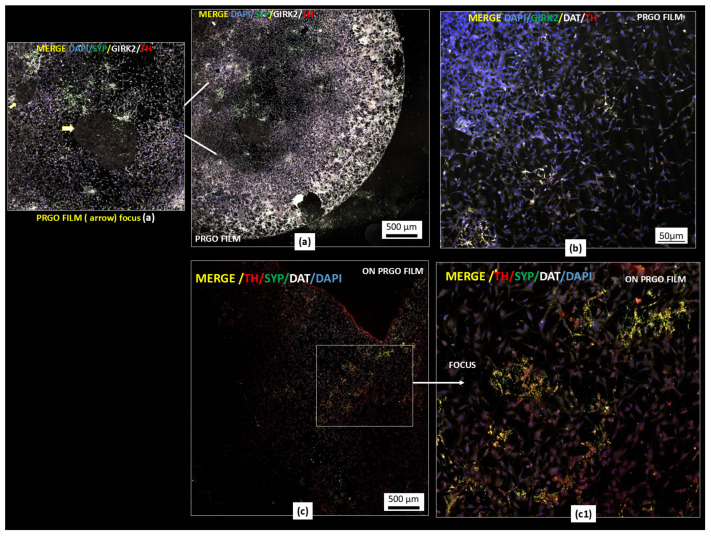
Dopaminergic maturation markers in GM-treated cells. PRGO film cultures show a portion of cover slide microscope (4 mm) TH (red)/GIRK2 (white)/synaptophysin (green)/DAPI (blue) (**a**) and TH (red)/DAT (white)/GIRK2 (green)/DAPI (blue) (**b**) and TH (red)/DAT (white)/SYP (green)/DAPI (blue) (**c**,**c1**). Scale bars: 500 µm (**a**,**c**); 50 µm (**b**). Representative images are presented; magnification: objective Pla-apochromat 20× 0.8M27 scanned area (specification in Methodology section).

Quadruple immunostaining (Figure 3) for GIRK-2 (a marker of dopaminergic maturation), DAPI (nuclear marker), synaptophysin (synaptic marker), tyrosine hydroxylase (TH, dopamine synthesis enzyme), and DAT (a dopamine transporter) revealed distinct cellular organization.

Regarding the interconnection in clustered SN4741 cells, the forming networks are displayed in Figure 3a. The cells formed interconnected networks on the PRGO film, with merged signals appearing to be white/pink depending on the TH expression levels. Figure 3b confirms the staining specificity, where positive and negative cells are shown for quadruple markers alongside the previously described intercellular contacts. Higher-resolution imaging highlighted the cellular organization on PRGO film-treated cultures (Figure 3c).

### 2.2. Neuroprotective Potential of Graphenic Materials Against MPP^+^-Induced Mitochondrial Dysfunction and α-Synuclein Accumulation

**Figure 4:**
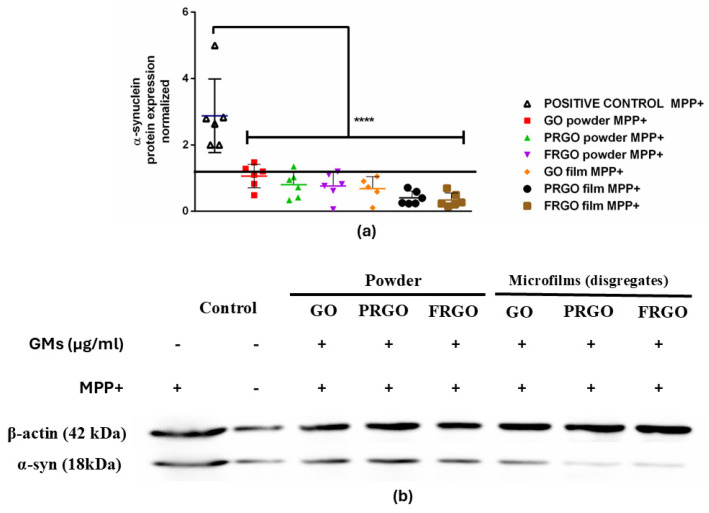
α-Syn expression in MPP^+^-treated cells. (**a**) Dot plots of normalized α-Syn levels (C+: MPP^+^; C−: untreated). Data were analyzed using one-way ANOVA followed by Dunnett’s multiple comparison test. Dot plots show significant statistics: **** *p* < 0.0001 vs. (**b**) immunoblot of α-Syn (18 kDa band) and β-actin (42 kDa) in SN4741 cells treated with 400 µM of MPP^+^ for 24 h (n = 6).

Given the central role played by α-Syn in α-synucleinopathies and PD, we further evaluated the protein levels of α-Syn in cultures treated with GMs alone or combined with MPP^+^. Our results showed that PRGO and FRGO films (50 μg/mL) significantly decreased α-Syn levels (* *p* < 0.05) after 7 days of treatment (Figure 4).

**Figure 5:**
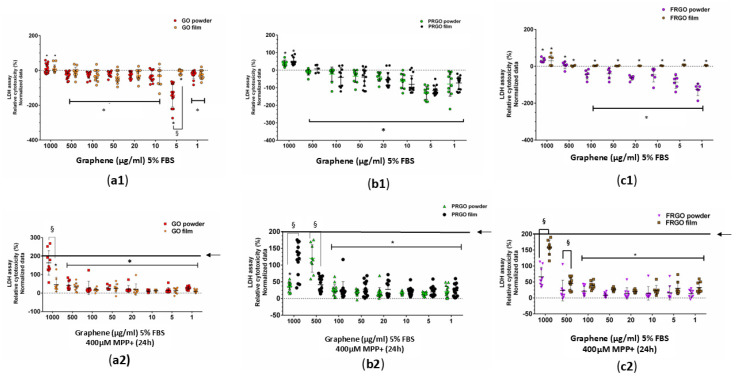
Cytotoxicity assessment using an LDH assay. SN4741 cells were treated for 7 days with GMs, with data normalized to control cells (no treatment, C−). Statistics show significance compared to negative control samples (C−). (**a1**–**c1**) show cells with GMs. Cells with the MPP^+^ treatment are shown in (**a2**–**c2**), which show the difference between the positive control (C+; MPP^+^ treatment, 200% LDH release, indicated by a black line and arrow) and the GM samples. Data were analyzed using one-way ANOVA followed by Tukey’s multiple comparison test. Dot plots show significant statistics: * *p* < 0.05 vs. C+ (no significant differences vs. C− in (**a2**–**c2**); statistical significance: § *p* < 0.05 between powder and film for the same GM species (n = 3).

Exposure to MPP^+^ (400 μM) increased the levels of α-Syn threefold (Figure 4b), an effect that was effectively prevented by all forms of GMs (* *p* < 0.001). Specifically, the PRGO and FRGO films reduced the toxicity of α-Syn to approximately 50% of the negative control (untreated cells without MPP^+^). We next examined whether graphenic materials (GMs) could attenuate the neurotoxic effects of MPP^+^, a dopaminergic neurotoxin known to induce mitochondrial dysfunction. Cell damage was assessed by measuring the lactate dehydrogenase (LDH) released in cells cultured for 7 days at 5% FBS (* *p* < 0.05 vs. C-negative). In the absence of MPP^+^, dispersed GMs significantly reduced LDH release, with the most pronounced effect observed at concentrations of 5 and 1 μg/mL. Treatment with MPP^+^ (400 μM) for 12 h increased cytotoxicity to 200% compared to the control (statistically significant, shown in Figure 5 as § with *p* < 0.05 C+ vs. samples). Notably, higher GM concentrations (500–1000 μg/mL) were less effective at mitigating LDH release than lower concentrations (1–100 μg/mL) (Figure 5(a1–c1)).

### 2.3. Neuroprotective Effects of GMs Against Tunicamycin-Induced Endoplasmic Reticulum Stress via Modulation of the Pre-Adaptive Stress Response

**Figure 6:**
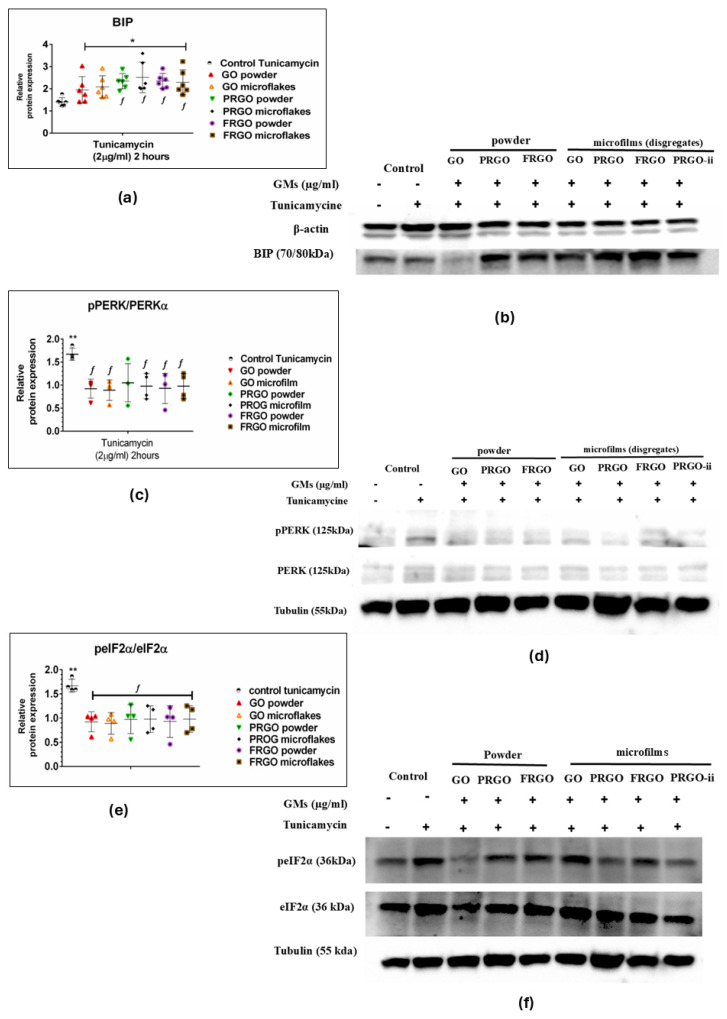
ER stress response proteins. Immunoblots and dot plots of BIP-GRP78 (78 kDa) (**a**,**b**), pPERK/PERK (150 kDa) (**c**,**d**), peIF2α/eIF2α (36 kDa) (**e**,**f**), and loading controls β-actin (42 kDa) and tubulin (55 kDa). Data were analyzed using one-way ANOVA followed by Dunnett’s multiple comparison test. Dot plots show significant statistics: * *p* < 0.05, ** *p* < 0.01 vs. C−; *f p* < 0.05 vs. Tunicamycin-treated controls (C+) (n = 6).

The results for the ER stress induction via Tunicamycin exhibited a significant increase in the chaperone BIP-GRP78 as a response to the cellular adaptation to Tunicamycin-induced stress (Figure 6a,b). Specifically, GMs increase the relative expression of BIP with an average fold change of 2 (* *p* < 0.05) compared to the untreated control samples. However, a notable observation is that the increase in BIP-GRP78 is significantly higher in PRGO species, comprising both powder and microflakes, compared to the positive control (control—Tunicamycin), achieving up to a 3.5-fold increase in relative BIP-GRP78 expression (*f p* < 0.05) in the PRGO microflakes. Similarly, the FRGO powder and microflake species also show a significant 2.2-fold increase. Regarding the activation of the UPR complex as an ER stress sensor PERK/P-PERK (Figure 6c,d), the results show a significant reduction in PERK phosphorylation in almost all GM species (and a tendency to reduce in PRGO powder) compared to the positive control (control—Tunicamycin), which increases sensor phosphorylation 1.6-fold compared to the GO species (powder and microflake), PRGO microflakes, and FRGO (powder and microflakes) of the pPERK/PERK ratio, which implies no activation of UPR stress signaling.

Nevertheless, our results show that the negative control (cells cultured without GM or Tunicamycin treatment) was significantly lower (* *p* < 0.001) than cells treated with only Tunicamycin (positive control); there was no change since cells treated with GMs maintained conditions equivalent to the negative control. Likewise, the analysis of eIF2α activation (Figure 6e,f) through its phosphorylation (p-eIF2α) showed a significant decrease compared to the positive control (control—Tunicamycin), showing a twofold increase in the p-eIF2α ratio compared to GM species. As with the p-PERK/PERK sensor, a significant increase in the positive control compared to the negative control (* *p* < 0.001) is observed, with no significant changes in GM treatments.

### 2.4. Exploring the Role of Torsin 1A in Tunicamycin-Induced ER Stress and the Modulatory Effects of GMs

**Figure 7:**
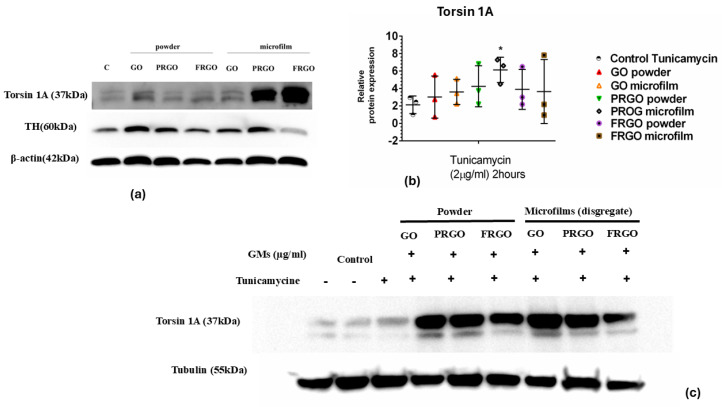
Torsin 1A expression under ER stress. (**a**) Immunoblots of Torsin 1A (37 kDa); TH (60 kDa) and b-actin (42 Kda) (42 kDa) in negative control cells (without GMs) and in GMs cells. (**b**) Data were analyzed using one-way ANOVA followed by Dunnett’s multiple comparison test. Dot plots show significant statistics: * *p* < 0.05 vs. Tunicamycin. (**c**) Immunoblots of Torsin 1A (62 kDa) and tubulin (55 kDa) in Tunicamycin-treated cells (n = 3).

Regarding the potential role played by Torsin 1A in ER stress, as a member of the AAA+ ATPase protein family that plays a critical role in ER function and the ER stress response [21,22], we investigated whether Torsin 1A could be relevant in terms of GMs and the prevention or attenuation of ER stress (Figure 7).

Our data from the immunoblot analysis of Torsin 1A revealed that samples treated with PRGO microflakes showed a fivefold increase (* *p* < 0.05) compared to the positive control samples (Figure 7b).

### 2.5. Analysis of cFos Response Concerning MPP^+^-Induced Oxidative Stress and the Protective Effects of GMs

**Figure 8:**
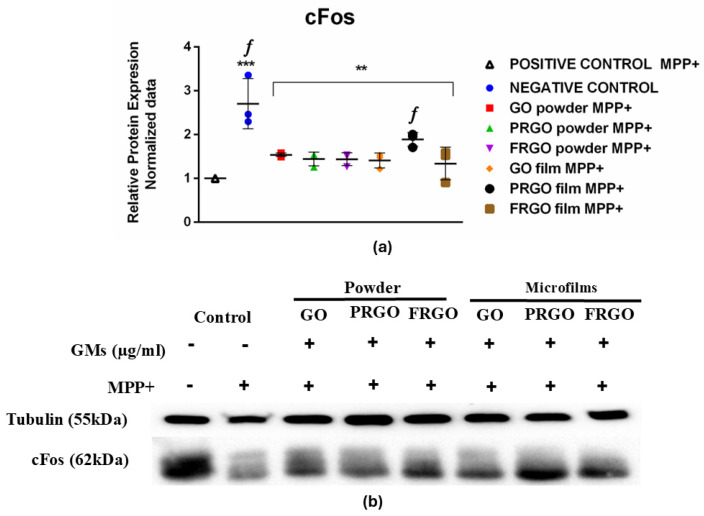
cFos expression in MPP^+^-treated cells. (**a**) Data were analyzed using one-way ANOVA followed by Dunnett’s multiple comparison test (n = 3). Dot plots show significant statistics: ** *p* < 0.01 vs. C−; *** *p* < 0.001 vs. C− (untreated) and significant statistic *f p* < 0.05 vs. C+ (MPP^+^), with PRGO microflakes exhibiting significantly higher cFos levels. (**b**) Immunoblots of cFos (62 kDa) and tubulin (55 kDa).

Our results (Figure 8) showed a significantly elevated cFos signal compared to the positive control (MPP^+^, * *p* < 0.0001; *f p* < 0.05) and MPP^+^-treated cells exposed to GMs. A significant reduction (2.8-fold) in cFos levels was observed in the MPP^+^-positive control. In contrast, the results for the cells treated with GMs and MPP^+^ showed increased cFos synthesis, which was significantly pronounced in the PRGO microflakes (twofold in comparison with C+, *f p* <0.05).

## 3. Discussion

### 3.1. Dopaminergic Maturation

Consistent with our prior findings [27], we found that GMs affect neuronal maturation, revealing significant morphological changes in cells following long-term treatment compared to the untreated controls. While the control cells retained a fibroblastic morphology, proliferated extensively until confluence was reached, and eventually detached and died following long-term culture, the GM-treated cells remained viable and adherent, showing morphological changes indicative of a differentiation process. Epifluorescence imaging using Hoechst 33258 staining confirmed robust cellular adhesion to GM scaffolds. These results are consistent with emerging evidence that graphene-based substrates enhance neural network development by combining excellent electrical conductivity, mechanical flexibility, and surface properties conducive to neurite outgrowth and synaptic formation [29,30]. Notably, cells not only adhered to the external surfaces of the GMs but also intercalated within the three-dimensional architecture of the dispersed material, indicating favorable topographical and biochemical interactions that facilitate integration. This behavior aligns with previous reports showing that graphene substrates provide biocompatibility and promote cell anchoring through enhanced protein adsorption and surface roughness [30,31]. Among the tested materials, PRGO films stood out by supporting complex, three-dimensional neural network formation. Critically, we observed a marked upregulation of tyrosine hydroxylase (TH) in Tuj-1/TH double-positive neurons, indicating not only neuronal differentiation [32] but also commitment toward a dopaminergic phenotype—a key requirement for applications in Parkinson’s disease (PD) modeling and cell replacement therapies [33]. Notably, PRGO film cultures exhibited a complex three-dimensional structural organization, with the marked upregulation of TH in Tuj-1/TH double-positive cells, thereby underscoring its efficacy to drive dopaminergic lineage commitment, consistent with our earlier RT-PCR data showing the upregulation of early dopaminergic lineage markers such as *Pitx3*, *Lmx1a*, and *Lmx1b* [25]. The sustained expression of mature dopaminergic markers, including DAT and GIRK2, further supports the functional maturation of these neurons. The presence of DAT is especially significant, as it is a definitive marker of dopaminergic identity and synaptic functionality. One of the most compelling findings of this study is the emergence of network-like structural organization in PRGO cultures, suggestive of functional neuronal connectivity. This observation is further supported by immunoblot and immunofluorescence data showing widespread distribution of SYP, a presynaptic vesicle protein, alongside TH and DAT across the PRGO surface [25]. The colocalization and spatial integration of these markers indicate the formation of synapse-like structures and suggest that PRGO films support neuronal survival and differentiation and foster functional network assembly. Our data extend these findings by showing that PRGO films can support individual neuronal differentiation and the emergence of cohesive, dopaminergic-enriched networks—a critical step toward engineering functional neural tissues [25,34].

### 3.2. Neuroprotective Effect at Low-GM Concentrations

The LDH results showed that GMs likely exhibit a cytotoxic effect at high concentrations, as previously reported by other authors [17,35], highlighting the importance of determining the optimal dosimetric concentration as a biologically suitable parameter. In addition, reducing the release of LDH in low-GM concentrations within the 1–100 μg/mL range could be shown as a characteristic of protecting dopaminergic neurons from the oxidative stress induced by MPP^+^. In summary, these findings underscore the neuroprotective potential of GMs in reducing dopaminergic neurotoxicity and lowering the α-Syn accumulation induced by MPP^+^, which is consistent with the observed effects upon the release of LDH (Figure 5). One of the most important aspects to consider in reparative or regenerative medicine is the minimum dose needed to stimulate an effect without causing cellular toxicity [36]. Therefore, the effectiveness of low doses of growth factors (GMs) is significant for promoting neuroprotective or regenerative effects in a cytotoxic environment, such as in Parkinson’s disease (PD).

### 3.3. Oxidative Response of MPP^+^ in SN4741 with GMs

In this study, we investigated the role of GMs as pre-adaptive and protective factors against two types of cellular stress: oxidative stress mediated by MPP^+^, a neurotoxin that induces mitochondrial dysfunction [37]; and ER stress induced by Tunicamycin [9,38]. In SN4741 cells exposed to MPP^+^ [39], a significant increase in LDH release and α-Syn accumulation, key markers of cellular damage associated with Parkinson’s disease pathogenesis, was observed [38]. However, treatments with GMs, particularly those that functionalize PRGO, effectively mitigated these effects, reducing cellular toxicity by up to 50% compared to the positive controls. This could suggest that they are able to modulate mitochondrial homeostasis and prevent pathological α-Syn aggregation [24,40], a finding that correlates with the significant reduction in α-Syn in GM-treated cells despite exposure to cellular stress. Additionally, GMs demonstrated a notable dose-dependent capacity to decrease LDH levels at low concentrations (between 1 and 10 µg/mL), minimizing the risk of cytotoxic effects associated with higher exposures, thus highlighting the importance of optimizing dosing conditions to maximize therapeutic efficacy [35,36].

### 3.4. GM Maturation Related to Capacity to Respond to Stress Induced by Tunicamycin

When studying ER stress induced by Tunicamycin [40,41], partial activation of the complete UPR pathway was observed [42], with a significant increase in the expression of the chaperone protein BIP/GRP78 in SN4741 cells treated simultaneously with this stressor and GMs. Previous studies have also described a process of partial inhibition guided by molecules aimed at modulating ER stress in Parkinson’s models [43,44]. The action of Tunicamycin on cells induces ER stress by inhibiting N-linked protein glycosylation, which leads to an accumulation of misfolded proteins, which, in turn, triggers an increase in the levels of BIP-GRP78 chaperone to compensate for the stress levels [45]. Upon dissociating from the PERK sensor (PKR-like ER kinase), the chaperone BIP allows for its activation and phosphorylation of PERK to P-PERK (Figure 6). The overexpression of BIP/GRP78, a central protein in the response to misfolded proteins, indicates a preventive mechanism against toxin-mediated cellular stress, as previously described in other studies [46]. This effect is not only linked to cell survival processes and adaptive responses to oxidative stress but also to cellular differentiation and mitotic transition processes [47].

In this context, a significant correlation was observed between exposure to PRGO film and FRGO film and the loss of the neuroblast marker DCX, suggesting a GM-guided neuronal maturation process [47]. This phenomenon was corroborated by the coexpression of specific markers of mature dopaminergic neurons, including TH, dopamine transporter (DAT), GIRK2, synaptophysin, and DAPI, indicating robust dopaminergic functional activity. The coexpression of synaptophysin with TH and GIRK2 further supports the hypothesis that GMs promote the functional maturation of these neurons, enabling them to establish synapses and modulate dopaminergic signaling, a finding previously described and corroborated by our data [25]. In brief, these results suggest that the activation pathway of the UPR signaling cascade in response to ER stress appears to confer enhanced cellular protection, especially in PRGO. This protective mechanism seems to involve the upregulation of BIP-GRP78 synthesis, pre-adapting the cell to a stress condition induced by Tunicamycin. Another finding that reinforces the potential neuroprotective effect of PRGO is the significant increase in Torsin 1A mediated by Tunicamycin [6,9]. In summary, these findings suggest a pre-adaptive response of GMs to toxin-mediated ER stress, wherein the stressed cell appears to mitigate the toxin’s effects by increasing the synthesis of Torsin 1A [48]. This response aims to correct the folding defects induced by Tunicamycin.

### 3.5. Pre-Adaptative Response to Stress: A Potential Role of cFos

cFos is a type of IEG transcription factor [49] characterized by the cellular/trans-synaptic activations necessary for cellular adaptation. It is used to localize active neurons as it significantly increases in response to external stimuli. Although cFos has dual kinetics, it was reported that the initial response to its activation showed an increase in expression following a decrease in the cFos signal [50]. Another relevant finding was the significant increase in the expression of cFos [19,51] in samples treated with MPP^+^ and PRGO microflakes. In untreated control cells (negative control), the elevated cFos signal reflects basal protein conditions, indicating responsiveness to cellular activity [52]. These levels are associated with normal homeostatic processes and key physiological functions, such as regulating energy metabolism [28,53]. The increase in c-Fos synthesis in stressor conditions [54], which is significantly pronounced in PRGO microflakes in comparison with positive control cells, suggests a pre-adaptive response and potential protective effect of these materials against cellular stress [54,55]. This protective effect is further evidenced by the twofold upregulation of c-Fos in PRGO-microflake-treated cells compared to the MPP^+^-treated controls (C+), and this was found to be statistically significant (* *p* <0.05). These findings could indicate that PRGO microflakes may mitigate MPP^+^-induced stress by modulating activity-dependent neuronal plasticity pathways [48,56]. This increment, combined with a positive tendency in other GMs, suggests that these materials could promote neuronal partial activation and synaptic plasticity. The induction of cFos is well documented as a marker of neuronal activity in response to specific stimuli [55], and in the context of dopaminergic cells, it reflects the activation of signaling pathways related to cellular maturation and function [57]. In our previous studies, we found no significant variation in the levels of cFos under the toxic effect of rotenone, although there was a non-significant upward tendency in GM microflakes. However, treatment with MPP^+^ modified this tendency, highlighting the fact that cFos plays a potential role in response to mitochondrial oxidation [49,58]. Furthermore, studies in the literature indicate differences in cellular response mechanisms to oxidative stress induced by rotenone or MPTP (the ionized form MPP^+^) [56], and our research line similarly reflects subtle differences in reactions to these stressors in SN4741 dopaminergic cells [59]. Our immunofluorescence data support this hypothesis by demonstrating the coexpression of synaptophysin with DAT, TH, and GIRK2 in PRGO-film-treated samples, indicating the functional maturation of these neurons.

## 4. Materials and Methods

### 4.1. GM Production

Graphenic materials were synthesized by Abalonyx AS in Oslo, Norway using a production process developed by Rune Wendelbo, based on a modified version of the “Hummers method” [60] as previously described [25,56,61]. To produce the GMs, natural graphite powder was first oxidized with potassium permanganate serving as the oxidizing agent. The resulting GO was washed with HCl to prevent the precipitation of manganese as MnO_2_ and then centrifuged to form an aqueous paste. This paste was mixed with NaCl particles and dried into films. Dissolution of the NaCl yielded a GO scaffold. The films were either used without further heat treatment to produce GO film or subjected to thermal treatments: heating to 300 °C in an Ar atmosphere to obtain partially reduced GO (PRGO), or heating to 1100 °C in Ar to obtain fully reduced GO (FRGO). These materials were produced in both powder (GO, PRGO, FRGO) and film (GO, PRGO, FRGO) forms. As previously described [5,6,54], the samples were analyzed for their chemical composition using X-ray photoelectron spectroscopy analysis, high vacuum and conventional methods, and environmental scanning electron microscopy for the structure analysis at INA (Zaragoza, Spain).

### 4.2. Exposure of SN4741 Cells to GO, PRGO, and FRGO in Both Powder and Film Forms

The morphological changes in SN4741 cells cultured under normal conditions were analyzed using DMEM supplemented with 10% fetal calf serum (FCS), 0.6% glucose, penicillin–streptomycin (50 U/mL), and 2 mM L-glutamine, and these cells were maintained in a humidified atmosphere of 5% CO_2_. The medium was replaced every 4 days. The cells were then collected, trypsinized in Ca^2+^/Mg^2+^-free MEM containing 0.1% trypsin and 0.02% EDTA, and transferred to Ca^2+^/Mg^2+^-free HBSS containing 10% FBS as described by Son et al. (1999) [62]. The cells were cultivated at a density of 5.3 × 10^3^ cells/cm^2^ in complete medium (on sterile coverslips in a 48-well plate) (Labclinics SL Barcelona, Spain #PLC20009, and Thermo Fisher Scientific Inc.Waltham. Massachusetts, USA) exposed to the GMs, including GM powder forms (GO, PRGO, FRGO) and GM film forms (GO, PRGO, FRGO). The films were mechanically triturated and utilized as microflakes, with surface areas ranging from 5 to 10 µm^2^ at a concentration of 50 µg/mL. For maturation analysis using DA-mature markers (DAT; GIRK2;SYP; TH), the cells with GMs were cultured long term (for 30 days) in culture conditions as previously described [25,59]. Hoechst 33258 staining [63] was used to observe the nuclear morphology in live or fixed cells, enabling the discrimination of apoptotic nuclei. Images of primary maturation markers were captured after two weeks of treatment, and the resulting morphological alterations were analyzed by comparing the treated cells with SN4741 cells cultured under identical conditions but without exposure to GO. The morphological evolution of the cells was monitored using an inverted microscope (Nikon TE2000-U, Minato, Tokio, Japan) equipped with a Nikon DS5MC (Minato, Tokio, Japan) camera and a Zeiss LSM700 (Oberkochen, Germany) confocal microscope.

### 4.3. LDH Assay: Study of Neuroprotective Capacity of GMs Against MPP^+^ Treatment

To assess the potential neuroprotective action of GO, PRGO, and FRGO (powder/microflakes) in SN4741 cells, these cells were treated with MPP^+^ (Sigma-Aldrich, Saint Louis, MO 63103 United States) under conditions previously established for SN4741 cell culture [13,64]. SN4741 cells were seeded at 3.3 × 10^3^ cells/cm^2^ for 7 days in a 96-well culture plate (Corning, Somerville, Massachusetts, USA) and incubated under normal conditions for 24 h to ensure cellular stabilization. Subsequently, the medium was replaced with essential medium (DMEM) without phenolphthalein in 5% FCS as previously described [24,25,59] and containing varying concentrations of GMs. The concentrations tested for each of the GMs (powder/microflakes: GO; PRGO; FRGO) were as follows: 1 mg/mL, 0.5 mg/mL, 0.1 mg/mL, 0.05 mg/mL, 0.02 mg/mL, 0.01 mg/mL, 0.005 mg/mL, 0.001 mg/mL, and 0 mg/mL (negative control). The cytotoxic effects of the GMs, GO, PRGO, and FRGO, in both powder and film forms, on the SN4741 cells (seeded at an 3.3 × 10^3^ cells/cm^2^ for 7 days in a 96-well culture plate (Corning) and exposed to different types of GMs) were analyzed at concentrations of 0, 1, 5, 10, 20, 50, 100, 500, and 1000 µg/mL in essential medium (DMEM) without phenolphthalein in 5% FCS/FBS as previously described [25,59]. On the seventh day of GM treatment, the cells were exposed to MPP^+^ (400 µM) for 24 h to induce oxidative stress, as previously described [64], which reduced the viability of the SN4741 cells. The recovery or protection against MPP^+^ treatment was measured using the LDH assay. All experiments were conducted using untreated cells as negative controls and cells treated with 10% Triton X-100 and MPP^+^ as positive controls. The LDH data were relative to c-cells. The analysis was performed as previously described [59,64] and was carried out by quantifying the release of the intracellular enzyme lactate dehydrogenase (LDH, EC 1.1.1.27 #11644793001, Merk, Burlington, Massachusetts, USA).

### 4.4. Western Blot Analysis of Proteins

SN4741 cells treated with GMs and controls were cultured in 60 mm well plates under various experimental conditions for designated time intervals. The cells were washed twice with PBS and lysed using 100 µL of RIPA buffer containing protease inhibitors (1% NP-40, 0.5% sodium deoxycholate, 0.1% SDS, 100 µg/mL of PMSF, 30 µL/mL of aprotinin, and 1 mM of sodium orthovanadate). The lysate was scraped, transferred to microcentrifuge tubes, passed through a 21-gauge needle, and centrifuged at 16,000 rpm for 20 min at 4 °C. The supernatant was collected as total cell lysate. The protein samples (20 µg) were heated at 95 °C for 5 min in 1× Laemmli buffer and analyzed using 10% sodium dodecyl sulfate–polyacrylamide gel electrophoresis (SDS-PAGE) at 15 V/cm for 1 h. The proteins were transferred to a PVDF membrane for immunoblotting and were blocked for 4 h at room temperature (RT) in blocking buffer containing 5% non-fat dry milk in TBS buffer (0.1% Tween-20 in 0.1% TBS). The blot-stripping and reprobing buffer used was Restore™ PLUS Western Blot Stripping Buffer (#46430, Thermo Fisher Scientific, Rockford, IL, USA). The primary antibodies were incubated overnight at 4 °C, as described by Beauvais et al. [9], including the following: polyclonal rabbit anti-BIP (70/80 kDa, clone C50B12 #3177s), polyclonal rabbit anti-p-PERK (150 kDa, Thr980 clone 16F8), and polyclonal rabbit anti-PERK (150 kDa, clone C33E10) from Cell Signaling (Danvers, MA, USA); polyclonal rabbit anti-p-EIF2α (36 kDa, clone Ser51), monoclonal mouse anti-β-actin (ab6276), b-tubulin III/Tuj1 (ab78078), and polyclonal rabbit anti-Torsin 1A (37 kDa, ab34540) from Abcam (Cambridge, UK); polyclonal rabbit anti-EIF2α (36 kDa, sc-1138) and monoclonal mouse cFos (62 kDa, clone sc-271243) from Santa Cruz Biotech (Santa Cruz, CA, USA); and α-synuclein (α-Syn, monoclonal clone syn S211, Thermo-Fisher, band of around 18 kDa and predicted to identify a band of 14.5 kDa) as previously described [26]. Protein detection was performed using primary antibodies (1:1000 dilution) and secondary antibodies (1:10,000 dilution) in blocking buffer. Secondary goat antirabbit and mouse labeled horseradish peroxidase (HRP) antibodies (#111-035-144 and 115-035-003, respectively) were obtained from Jackson Laboratories (West Grove, PA, USA). Signals were detected using an enhanced chemiluminescence (ECL, SuperSignal West Dura substrates #34076) system (Thermo Fisher Scientific, Rockford, IL, USA), visualized with the ChemiDoc MP Imaging system from Bio-Rad. Quantification and normalization were completed using Fiji-ImageJ software (version Image 1.54p) as described previously [9,25,59].

### 4.5. Immunostaining Assay

SN4741 cells treated with GMs for 7 days (control cells without GMs) and 15 days and 30 days in culture with GMs, along with untreated controls, were fixed with 4% paraformaldehyde (*w*/*v*) in 0.1 M PBS (pH 7.4) for 20 min, rinsed three times with PBS, and permeabilized with 0.1% Triton X-100 in PBS. The cells were blocked with 5% normal goat serum (NGS, Invitrogen, Thermo Fisher, Waltham, Massachusetts, USA) in PBS containing 3% bovine serum albumin (BSA, Invitrogen). Primary antibodies were diluted at 1:100 in PBS with 3% BSA and incubated overnight at 4 °C. The following primary antibodies were used: polyclonal rabbit anti-tyrosine hydroxylase (TH, #NB300-109 Novus Biologicals, Centennial, CO 80112, USA); mouse anti-Tuj-1 (Anti-βIII Tubulin, clone 5G8, Promega Alcobendas, Madrid, Spain); polyclonal goat anti-GIRK2 (G-protein-regulated inward-rectifier potassium channel 2 protein, #ab65096; Abcam, Cambridge, Reino Unido); polyclonal rat anti-DAT (dopamine transporter, #MAB369 Millipore, Burlington, Massachusetts, United States); polyclonal rabbit anti-neurofilament-H NEFH (#ab8135); polyclonal rabbit anti-neurofilament M, NEFM (#25805-1-AP, Proteintech, Planegg-Martinsried Germany); monoclonal mouse anti-Doublecortin (DCX, #ab18723; Abcam); Hoechst 33,258 (live cells, PubmedChen #329770334, Bethesda, MD USA); and DAPI (Sigma-Aldrich, Saint Louis, MO 63103 United States). After incubation, the cells were washed with PBS containing 0.1% Tween-20 for 1 h at RT. Secondary antibodies (Alexa Fluor 488 goat anti-mouse #115-545-166; Alexa Fluor 555 goat anti-rabbit# 711-565-152; Alexa Fluor 647 donkey anti-rat# 712-605-150, and anti-goat# 705-605-147; Jackson Laboratories) were diluted 1:500 in PBS with 3% BSA/blocking and incubated for 2 h at RT. The cells were washed for 1 h in PBS with 0.1% Tween-20, and nuclei were stained with DAPI (1:200, Sigma, Saint Louis, MO 63103 United States) for 15 min at RT. Colocalization analysis for fluorescence microscopy was performed using Fiji-ImageJ NIH [27,58], and images were obtained using a confocal microscope (LSM700, Zeiss; Zen2010B SP1 ZEN-2010B SP1 v. 6.0.0.485 software (Zeiss, Oberkochen, Baden-Württemberg, Germany). For the high-resolution image, tile scans were acquired using a laser scanning confocal microscope equipped with a motorized stage. To cover large tissue areas, a tile-scan mosaic was performed with 5 × 5 tiles, using an objective Pla-apochromat 20× 0.8M27 air objective (numerical aperture 0.8). The scanning area was defined manually (scan model: frame, Pixel Dwell 0.37µsec size 2048y*2048x; bip Depth 12 Bit) and images were acquired with a step size of 1 µm to capture the full depth of the sample; the scan area had an image size of 348.3 µm × 348.3 µm and a pixel size of 0.17 µm. Fluorescence signals were detected in sequential scanning mode to prevent channel crosstalk.

Statistical Analysis

The results are expressed as mean ± standard deviation (SD). Detailed information of the test used, including the independent repetitions of the experiment, specific post hoc tests, and *p*-values, is stated in the figure legends. Data were analyzed using GraphPad Prism software version 6.01 (GraphPad Software, San Diego, CA, USA). The normality of the data was assessed using the Shapiro–Wilk test for each variable. For the comparison of multiple groups, one-way ANOVA was used followed by Dunnett’s test or multiple comparison test using Tukey’s post hoc test. A *p*-value * < 0.05 was considered statistically significant.

## 5. Conclusions

The potential protective effect of GMs is evidenced by reductions in the release of lactate dehydrogenase (LDH), the accumulation of α-Syn, and markers of ER stress, suggesting that GMs may act through multiple pathways to preserve neuronal integrity. In summary, our findings reveal that GMs, particularly PRGO microflakes, upregulate the expression of BIP/GRP78 while preventing the activation of the UPR signaling cascade, thereby pre-adapting cells to stress conditions and enhancing their resilience to Tunicamycin-induced damage. In untreated control cells, elevated levels of cFos reflect basal activity and homeostatic processes, whereas the downregulation of cFos in response to MPP^+^ indicates severe cellular damage or metabolic dysfunction. Remarkably, GM-treated cells exhibit a tendency to increase cFos synthesis, suggesting a pre-adaptive response that may confer partial protection against oxidative stress. This effect is particularly pronounced in PRGO microflakes, underscoring their superior neuroprotective capacity compared to other GM variants. In summary, our results position GMs used at a low concentration, especially PRGO microflakes, as promising neuroprotective agents in the context of neurodegenerative diseases, especially those associated with oxidative stress and protein misfolding alterations, highlighting their potential to modulate key pathways such as the UPR and promote the functional maturation of dopaminergic neurons.

## Data Availability

The raw data supporting the conclusions of this article are included in an open database: https://doi.org/10.5281/zenodo.16993845 (accessed on 1 September 2025).

## Abbreviations

The following abbreviations are used in this manuscript:

GMs	graphenic materials
DCX	Doublecortin
ER	endoplasmic reticulum
FRGO	fully reduced graphene oxide
DAT	dopamine transporter
G	graphene
GIRK2	G-protein-regulated inward-rectifier potassium channel 2 protein
GO	graphene oxide
IEG	immediate early gene
LDH	lactate dehydrogenase
MPP^+^	1-methyl-4-phenylpyridinium ion
MTPT	1-methyl-4-phenylpyridinium
PD	Parkinson’s disease
PRGO	partially reduced graphene oxide
TH	tyrosine hydroxylase
Tuj-1	beta-III tubulin
UPR	unfolded protein response
α-Syn	α-synuclein
